# Growth and Stress Axis Responses to Dietary Cholesterol in Nile Tilapia (*Oreochromis niloticus*) in Brackish Water

**DOI:** 10.3389/fphys.2018.00254

**Published:** 2018-03-26

**Authors:** Chang Xu, Erchao Li, Zhixin Xu, Yujie Su, Minghui Lu, Jian G. Qin, Liqiao Chen, Xiaodan Wang

**Affiliations:** ^1^Department of Aquaculture, College of Marine Sciences, Hainan University, Haikou, China; ^2^School of Life Sciences, East China Normal University, Shanghai, China; ^3^Hainan Dingda Aquaculture Co., Ltd., Wenchang, China; ^4^School of Biological Sciences, Flinders University, Adelaide, SA, Australia

**Keywords:** cholesterol, HPI-axis, cortisol, Na^+^/K^+^-ATPase, salinity, Nile tilapia *Oreochromis niloticus*

## Abstract

Six isonitrogenous and isocaloric diets were formulated to contain 0% (control), 0.4, 0.8, 1.2, 1.6, or 2.4% dietary cholesterol and fed to juvenile Nile tilapia (*Oreochromis niloticus*) (2.20 ± 0.12 g) twice daily to apparent satiation for 8 weeks in triplicate at a salinity of 16. Fish fed 0.4% cholesterol showed a higher weight gain and specific growth rate and a lower feed coefficient ratio than fish fed other diets. No difference was found in the survival of Nile tilapia fed various levels of cholesterol. Cholesterol in the serum and liver and low-density lipoprotein cholesterol in the serum increased with the increase in the dietary cholesterol content. Relative to the control, no significant difference was found in the expression of head kidney P450scc mRNA between treatment groups. The expression of head kidney 11β-HSD2 mRNA was the highest in the control group, and it decreased significantly with increasing levels of diet cholesterol. Fish fed 0.4 or 1.2% cholesterol had a higher 20β-HSD2 mRNA expression in the head kidney than those fed other diets. Fish fed 0.8% cholesterol had higher expressions of GR1 and GR2B mRNA in the liver than other groups. Fish fed 0.4% cholesterol had the highest activity of gill Na^+^/K^+^-ATPase. Fish fed 0.8 to 2.4% cholesterol had higher serum cortisol contents than the fish in the control group and the fish fed 0.4% cholesterol. This study suggests that dietary cholesterol is not essential for Nile tilapia survival in brackish water, but 0.4% cholesterol supplementation in the Nile tilapia diet contributes to the improvement of hyperosmotic adaptation and increases in gill Na^+^/K^+^-ATPase activity and serum cortisol content by regulating the hypothalamic-pituitary-interrenal stress axis.

## Introduction

Cholesterol, as a main structural component of animal cell membranes and a precursor for biosynthesis of vitamin D3, prostaglandins, steroids and bile acids, is an essential nutrient for eukaryotic animals (Steffens, 1989; Fast and Boyd, 1992; Sheen et al., 1994). Since bony fish have the cholesterol synthesis ability, it is not essential to add dietary cholesterol for the health of bony fish (Sealey et al., 2001; Jobling, 2011). However, recent research indicates that cholesterol supplements to a fish diet formulated with soybean meal as a protein source can improve the growth performance of tongue sole (*Cynoglossus semilaevis*) larvae (Han, 2013) and promote the growth of channel catfish (*Ictalurus punctatus*) (Twibell and Wilson, 2004). In addition, the addition of 0.5–1.5% cholesterol to a flue-cured soybean meal diet can significantly improve the activity of digestive enzymes and promote digestion and absorption in Japanese flounder (*Paralichthys olivaceus*) (Chen, 2006). Dietary supplementation of 0.6–1.2% cholesterol can enhance the nonspecific immunity of rainbow trout (*Oncorhynchus mykiss*) (Long et al., 2013). However, our knowledge is limited on other physiological roles that cholesterol can play to improve fish health under stress conditions, although cholesterol is closely linked to fish stress through the hypothalamic-pituitary-interrenal axis (HPI axis) (Mormède et al., 2007).

**Figure 1:**
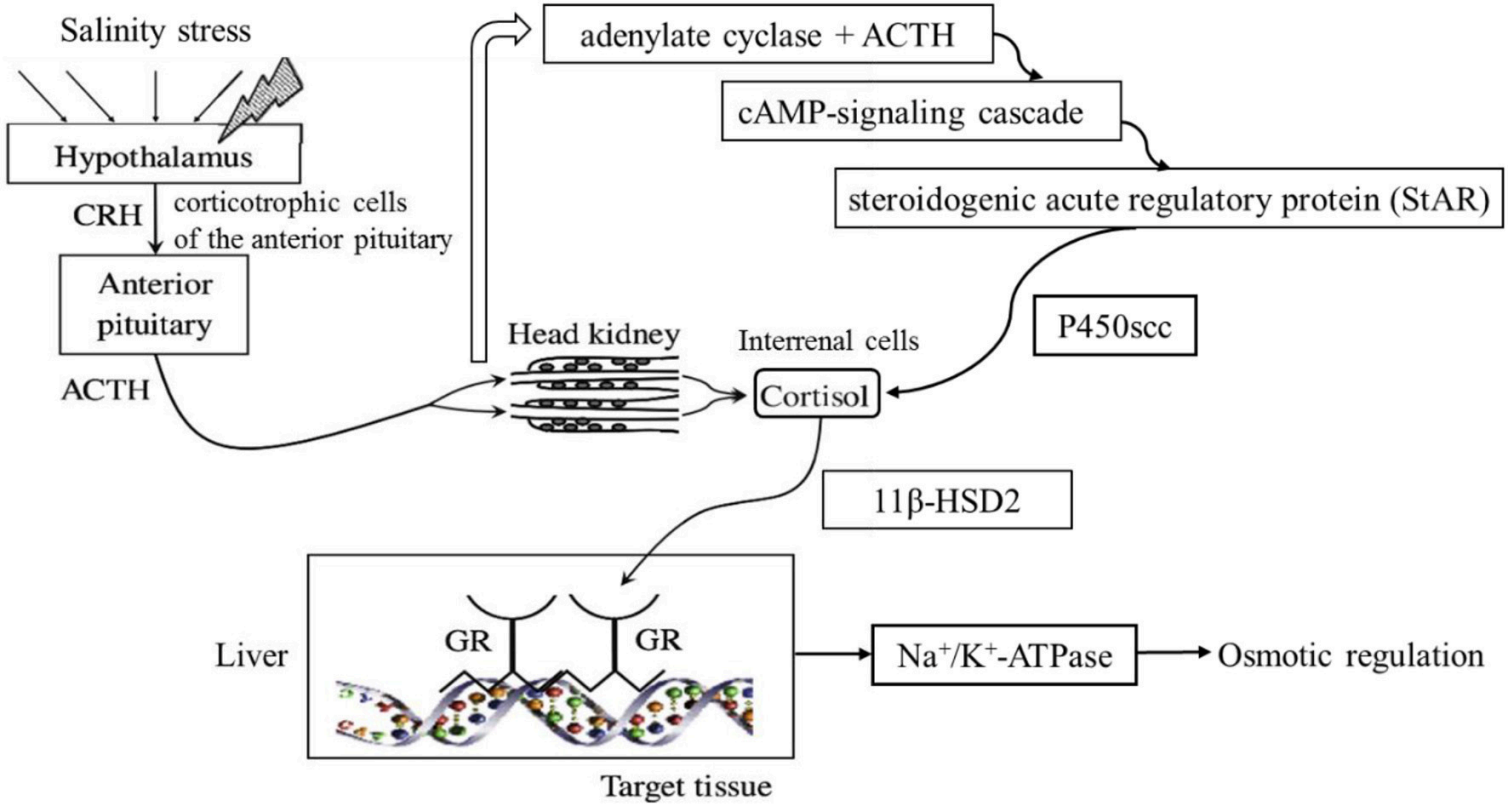
Conceptual pathways of the influence of dietary cholesterol. Upon exposure to a stressor, the hypothalamus secretes CRH in the area of the corticotropic cells of the anterior pituitary. In response, the corticotrophs secrete adrenocorticotropic hormone derived from proopiomelanocortin (ACTH) to the circulation, which stimulates cortisol synthesis and secretion from the interrenal cells of the head kidney in fish. The ACTH binding with adenylate cyclase stimulates the cAMP-signaling cascade. The StAR limits the transportation of cholesterol, and the P450scc restricts the conversion from cholesterol to cortisol. Cortisol binds to the GR and regulates the expression of Na^+^/K^+^-ATPase, which is involved in osmoregulation.

In bony fish, cortisol is a main hormone in the HPI axis that maintains the balance of physiological and biochemical processes when fish are under stress (Bonga and Wendelaar, 1997; Barton, 2002; Flik et al., 2006). When fish are stressed, the hypothalamus releases the corticosteroid-releasing factor (CRF), which promotes the secretion of an adrenocorticotropic hormone derived from proopiomelanocortin (ACTH) in the anterior pituitary gland (Huising et al., 2004; Metz et al., 2004). After entering the circulatory system, ACTH binds to the melanocortin 2 receptor (MC2R) for steroid synthesis in the stromal cells of the head kidney and promotes the conversion of cholesterol to cortisol (Aluru and Vijayan, 2006). The conversion of cholesterol to cortisol involves several key proteins and pathways in the renal tissue of the head kidney. Steroid acute regulatory protein (StAR) transports cholesterol to the mitochondrial inner membrane of the renal cells, and the cholesterol side chain cleavage enzyme, cytochrome P450 (P450scc), controls the conversion rate of cholesterol to cortisol (Engelhardt et al., 1985; Aluru and Vijayan, 2008). The function of cortisol is limited by the enzymes 11β hydroxysteroid dehydrogenase 2 (11β-HSD2) and 20β hydroxysteroid dehydrogenase 2 (20β-HSD2). In most animals, cortisol binds to the glucocorticoid receptor (GR) and regulates glucose metabolism, fat metabolism and the osmotic pressure balance (Gold et al., 2002; Ismaili and Garabedian, 2004; Peckett et al., 2011). In fish, cortisol can maintain the osmotic pressure balance through the regulation of Na^+^/K^+^-ATPase activity in the gill epithelial cells (Seidelin and Madsen, 1999). Based on a review of the HPI axis in zebrafish (Alsop and Vijayan, 2009), a hypothesized pathway illustrating the influence of dietary cholesterol on fish under hyperosmotic conditions is presented in Figure 1.

Nile tilapia (*Oreochromis niloticus*) is a global aquaculture species, and its farming in brackish water has received much attention in the past decade because proper salinity acclimation can improve its flesh texture and taste (Li et al., 2007). In the past, research has focused on the physiological adaptation to salinity changes in Nile tilapia strains after selection for salinity tolerance and transgenesis (El-Zaeem et al., 2011). However, a variety of challenges, such as a high mortality (Lemarié et al., 2004), slow growth (Ntabo, 2012), and changes in the composition of digestive proteases (Fang and Shufen, 1989) have impacted the industry of Nile tilapia farming in brackish water. The analysis of the whole transcriptome of the liver of Nile tilapia cultured at different salinities shows that steroid biosynthesis, steroid hormone biosynthesis and ovarian steroid biosynthesis pathways are up-regulated by the increase in the environmental salinity, and cholesterol is the shared substance among these biosynthesis pathways (Xu et al., 2015). However, the potential role of cholesterol in the diet of Nile tilapia in alleviating hyperosmotic stress in brackish water is not clear. Is cholesterol an effective substrate for osmoregulation in Nile tilapia under brackish water conditions through the regulation of the HPI axis? Meanwhile, is cholesterol an effective nutrient for improving the growth performance of Nile tilapia under hyperosmotic stress? These questions are both the focus of our current study.

## Materials and methods

### Experimental diets

Six isonitrogenous and isocaloric diets (one control diet vs. five test diets) were formulated to contain equal levels of proteins (34%), lipids (6%) and digestible energy (3.2 kcal/g) (Table 1). The ingredients were obtained from Sangon Biotech (Shanghai) Co. Ltd., and the cholesterol had > 99% purity (A610122-0250, Sangon Biotech Co. Ltd., Shanghai, China). The actual cholesterol content of each diet were 0.00 (control), 0.42, 0.79, 1.17, 1.61, and 2.38 g/kg, and contents were measured with a cholesterol detection kit (F002, Nanjing Institute of Biological Engineering, Nanjing, China). Diets were processed into 3-mm-diameter pellets, dried at room temperature to a moisture content of approximately 10%, ground and sieved to an appropriate size and stored at −20°C until use (Peres et al., 2003).

**Table 1:** Percent composition and estimated nutrient contents of experimental diets.

**Composition**	**Content (%)**					
Group	0	0.40%	0.80%	1.20%	1.60%	2.40%
Casein	32	32	32	32	32	32
Gelatin	8	8	8	8	8	8
Corn starch	33	33	33	33	33	33
Corn oil	6	5.6	5.2	4.8	4.4	3.6
Vitamin mix^a^	1	1	1	1	1	1
Mineral mix^b^	2	2	2	2	2	2
CMC	3	3	3	3	3	3
Cholesterol	0	0.4	0.8	1.2	1.6	2.4
Celufil	15	15	15	15	15	15
**ESTIMATED NUTRIENT (%)**						
Crude protein	38.4	38.4	38.4	38.4	38.4	38.4
Crude lipid	6	6	6	6	6	6

a *Vitamin premix, diluted in cellulose, provided the following in mg/kg: Vitamin A (500,000 IU/g), 8 mg; Vitamin D_3_ (1,000,000 IU/g), 2 mg; menadione, 10 mg; dl-alpha tocopherol acetate, 200 mg; thiamin, 10 mg; riboflavin, 20 mg; pyridoxine, 20 mg; d-calcium pantothenate, 200 mg; nicotinic acid, 150 mg; vitamin B12, 0.02 mg; biotin, 2 mg; inositol, 400 mg; choline chloride, 2,000 mg; L-ascorbyl-2-polyphosphate (15% vitamin C activity),100 mg*.

b *Mineral premix (g/kg mixture): CaCO_3_, 314.0 g; KH_2_PO_4_, 469.3 g; MgSO_4_·7H_2_O, 147.4 g; NaCl, 49.8 g; FeSO_4_, 10.0 g; MnSO_4_·H_2_O, 3.120 g; ZnSO_4_·7H_2_O, 4.670 g; CuSO_4_·5H_2_O, 0.620 g; KI, 0.160 g; CoCl_2_·6H_2_O, 0.080 g; NH_4_ molybdate, 0.060 g; NaSeO_3_, 0.020 g*.

### Experimental fish, feeding, and sampling

Sex-reversed all-male Nile tilapia (*O. niloticus*) were obtained from a local farm in Shenzhen, China, and reared in the laboratory for 2 weeks. 522 healthy juveniles (2.20 ± 0.12 g) were randomly divided into six groups and stocked in 18 white polyethylene tanks (500 L) with three replicates of 29 fish per tank. Fish were acclimated to the target salinity of 16 by adding seawater and regulating a salinity increase of 4 units per day. The freshwater was aerated thoroughly and double-filtered before use. Seawater was pumped from the sea and filtered before it was added to adjust the water salinity. The salinity of the experimental water was 16, and it was checked by a salinity meter (AZ Instrument, Taiwan) every day. During the acclimation and experimental periods, the photoperiod was 12:12 dark: light; the temperature was 32 ± 3°C; the pH was 7.3 ± 0.2; and the dissolved oxygen was maintained above 7.6 mg/L. One half of the water was exchanged daily with aerated water at salinity 16. Unfed feed and feces were removed daily with a siphon tube. The unfed feed was dried and weighed.

At the end of the 8-week experiment, all fish were counted and deprived of feed for 24 h before sampling. The growth performance-related parameters were measured as follows:

Survival (%) = (N_i_ − N_f_) / N_i_ × 100

Weight gain (WG, %) = (W_f_ − W_i_) / W_i_ × 100

Specific growth rate (SGR, % day^−1^) = [ln ^(Wf)^ − ln ^(Wi)^] × 100/days

Feed conversion ratio (FCR) = W_t_ / (W_f_ − W_i_)

N_i_ is the initial fish number, and N_f_ is the final fish number. W_i_ represents the initial average weight, while W_f_ is the final average weight. W_t_ indicates the amount of feed intake.

Eight fish from each tank were anesthetized before sampling in 3-aminobenzoic acid ethyl ester methane-sulfonate (MS-222, Sigma, USA) for physiological parameter measurements. Blood samples were taken from the fish caudal vein when there was no response to touch. Blood samples in centrifuge tubes stood overnight at 4°C and were centrifuged at 3,000 rpm for 5 min at 4°C (Eppendorf, Germany) before the serum was stored at −80°C. The liver, head kidney and the second gill arch of each fish were extracted and frozen immediately in liquid nitrogen and then stored at −80°C. All experiments were conducted under the standard protocols for the Care and Use of Laboratory Animals at East China Normal University (Animal ethics approval number: F20140101).

### Biochemical parameters assay

Nine samples of the serum, liver and gill tissues in each treatment (three per tank) were thawed on ice. The contents of cholesterol in the serum and liver were measured with a cholesterol detection kit (F002, Nanjing institute of Biological Engineering, China). The content of low-density lipoprotein cholesterol (LDL-C) was measured with an LDL-C detection kit (A113-1, Nanjing institute of biological engineering, Nanjing, China), as described by Nauck et al. (2002). The activity of Na^+^/ K^+^-ATPase was tested using a Na^+^/ K^+^-ATPase detection kit (A070-2, Nanjing institute of Biological Engineering, Nanjing, China) by detecting the amount of inorganic phosphate released from ATP (Bełtowski and Wójcicka, 2002).

### RNA extraction, cDNA synthesis and quantitative real-time PCR

Total RNA was extracted from the head kidney and liver using Trizol (RN0101, Aidlab, China) according to the manufacturer's protocol. The quality and quantity of the total RNA were measured using a Nano Drop 2000 spectrophotometer (Thermo, Wilmington, USA), and 1% agarose polyacrylamide gel electrophoresis was used to check RNA integrity. A PrimeScript™ RT reagent kit (RR036A, Takara, Japan) was used to synthesize the cDNA. The reverse transcription system contained 4 μL of 5 × PrimeScript RT Master Mix and 1 μg of total mRNA in a 20-μL volume made up with nuclease-free water. The reverse transcription protocol was conducted at 37°C for 15 min and 85°C for 5 s.

The six groups containing nine parallel samples and two technical repeats were run for each cDNA sample. The nucleic acid sequences of EF1α (AB075952.1), 20β-HSD2 (KM279628.1), 11β-HSD2 (AY190043.2), P450scc (FJ713103.1), GR1 (XM_013271702.1) and GR2B (AB245406.1) were downloaded from the National Center for Biotechnology Information (http://www.ncbi.nlm.nih.gov/) and used as samples to design the specific fluorescence quantitative primers with Primer 6.0 (Table 2). The 11β-HSD2, 20β-HSD2 and P450scc sequences in the head kidney and the GR1 and GR2B sequences in the liver were amplified in a 20 μL reaction volume containing 750 ng of cDNA, 0.4 μM of each primer, 10 μL of UltraSYBR mixture (CW0957, KangWei, China) and supplemental nuclease-free water by using a Bio-Rad CFX96 RealTime PCR system (BioRad, USA). The reaction program was as follows: 95°C for 30 s, followed by 39 cycles of 94°C for 15 s and 58°C for 20 s, and then a final step at 72°C for 20 s. The EF1A gene was used to normalize the target genes (Yang et al., 2013). The 2^−ΔΔCt^ method of processing data was used to obtain the fold change between control group and other groups (Schmittgen and Livak, 2008).

**Table 2:** Primers of genes in the HPI-axis used in this study.

**Primer**	**Sequence (5′ → 3′)**	**Production size (bp)**
11β-HSD2	CACCATCCTGCCATCATC	92
	CCTCACCGTAGTCCTCAA	
20β-HSD2	ACACATGGCTACAGTATCTAC	95
	ACAGTCAGTAGTGGTCATTC	
P450scc	GGAGGAGGATTGCTGAGA	118
	GGTGACTGTGGTGTTGAG	
GR2	CCGTTGAAGGATGGAGAG	97
	CAGGCTGGACAGTTCTTC	
GR1	CCTCTGCCTCTGTCATTG	98
	CGTCTGCGTCTGAAGTAA	
EF1α	GGACTGGCTTATGCTGATT	100
	ACTGAGAAGAGGCACTGT	

### Statistical analysis

All data are presented as the mean ± standard errors (Mean ± S.E.) and were subjected to one-way ANOWA analysis of variance (SPSS for Windows, version 11.5) to determine significant differences between treatments after the homogeneity test. Data as percentages and gene expression levels were arcsine-transformed and then checked for homogeneity before one-way ANOVA analysis was conducted. If a significant difference was identified, the differences between the means were compared by Duncan's multiple range tests. The significance level for differences was set at *P* < 0.05, and the extremely significant difference level was set as *P* < 0.01.

## Results

### Growth performance

The survival of the Nile tilapia ranged from 80 to 96.67% and was not affected by the dietary cholesterol levels (Table 3). Fish fed the 0.4% cholesterol diet had higher weight gain and specific growth rate than the fish fed other diets with various cholesterol levels (*P* < 0.05), but no significant differences in these parameters was found when they were compared to those of the control fish (*P* > 0.05). The control fish and the fish fed 0.4% cholesterol had the lowest feed coefficient ratios, but a significant difference was only found when they were compared with the ratio of fish fed 1.6% cholesterol (*P* < 0.05) (Table 3).

**Table 3:** Weight gain, survival, specific growth rate, and feed coefficient of Nile tilapia fed different experimental diets for 8 weeks.

**Treatments**	**Survival (%)**	**Weight gain (%)**	**Specific growth rate (%)**	**Feed conversion ratio**
0.0%	88.89 ± 4.01	736.35 ± 12.24^bc^	3.79 ± 0.03^bc^	1.34 ± 0.68^a^
0.4%	84.45 ± 1.11	798.41 ± 35.04^c^	3.92 ± 0.07^c^	1.32 ± 0.29^a^
0.8%	88.89 ± 2.94	634.41 ± 30.60^a^	3.56 ± 0.07^a^	1.52 ± 0.34^abc^
1.2%	84.44 ± 2.94	658.67 ± 33.80^ab^	3.62 ± 0.08^ab^	1.61 ± 0.14^bc^
1.6%	85.56 ± 2.22	620.67 ± 21.96^a^	3.53 ± 0.05^a^	1.62 ± 0.02^c^
2.4%	95.56 ± 1.11	653.33 ± 24.26^ab^	3.60 ± 0.06^ab^	1.37 ± 0.07^ab^
*P*-value	0.083	0.004	0.005	0.035

*Numbers in the same column with different superscripts are significantly different (P < 0.05)*.

*Values of survival, weight gain, specific growth rate and feed conversion ratios (mean ± S.E.) are the mean of three replicate tanks*.

### The content of cholesterol in the serum and liver or LDL-C in the serum

The contents of cholesterol and LDL-C in the serum and the content of cholesterol in the liver significantly increased with the increasing percentage of dietary cholesterol, and the fish fed 2.4% cholesterol had the highest contents of cholesterol and LDL-C in the serum (*P* < 0.01) (Table 4).

**Table 4:** The contents of cholesterol and LDL-C in the serum and liver of Nile tilapia fed different cholesterol percentage diets for 8 weeks.

**Treatment**	**Cholesterol in serum (mmol/L)**	**Cholesterol in liver (mmol/L)**	**LDL-C^1^ in serum (mmol/L)**
0	3.22 ± 0.17^a^	0.43 ± 0.02^a^	1.51 ± 0.65^a^
0.40%	6.45 ± 0.19^b^	0.96 ± 0.06^b^	4.95 ± 0.62^b^
0.80%	7.92 ± 0.74^c^	1.07 ± 0.19^b^	6.96 ± 0.13^b^
1.20%	8.94 ± 0.03^cd^	0.98 ± 0.07^b^	12.44 ± 1.29^c^
1.60%	10.63 ± 0.40^d^	0.83 ± 0.66^b^	13.28 ± 0.27^c^
2.40%	15.39 ± 0.48^e^	1.06 ± 0.32^b^	14.91 ± 1.96^c^
*P*-value	0.001	0.003	0.001

*Numbers in the same column with different superscripts are significantly different (P < 0.05)*.

1 *LDL-C:low-density lipoprotein cholesterol*.

*Values of cholesterol in serum and liver and LDL-C in serum (mean ± S.E.) are the mean of nine fish*.

### The mRNA relative expressions of key genes in the HPI axis

**Figure 2:**
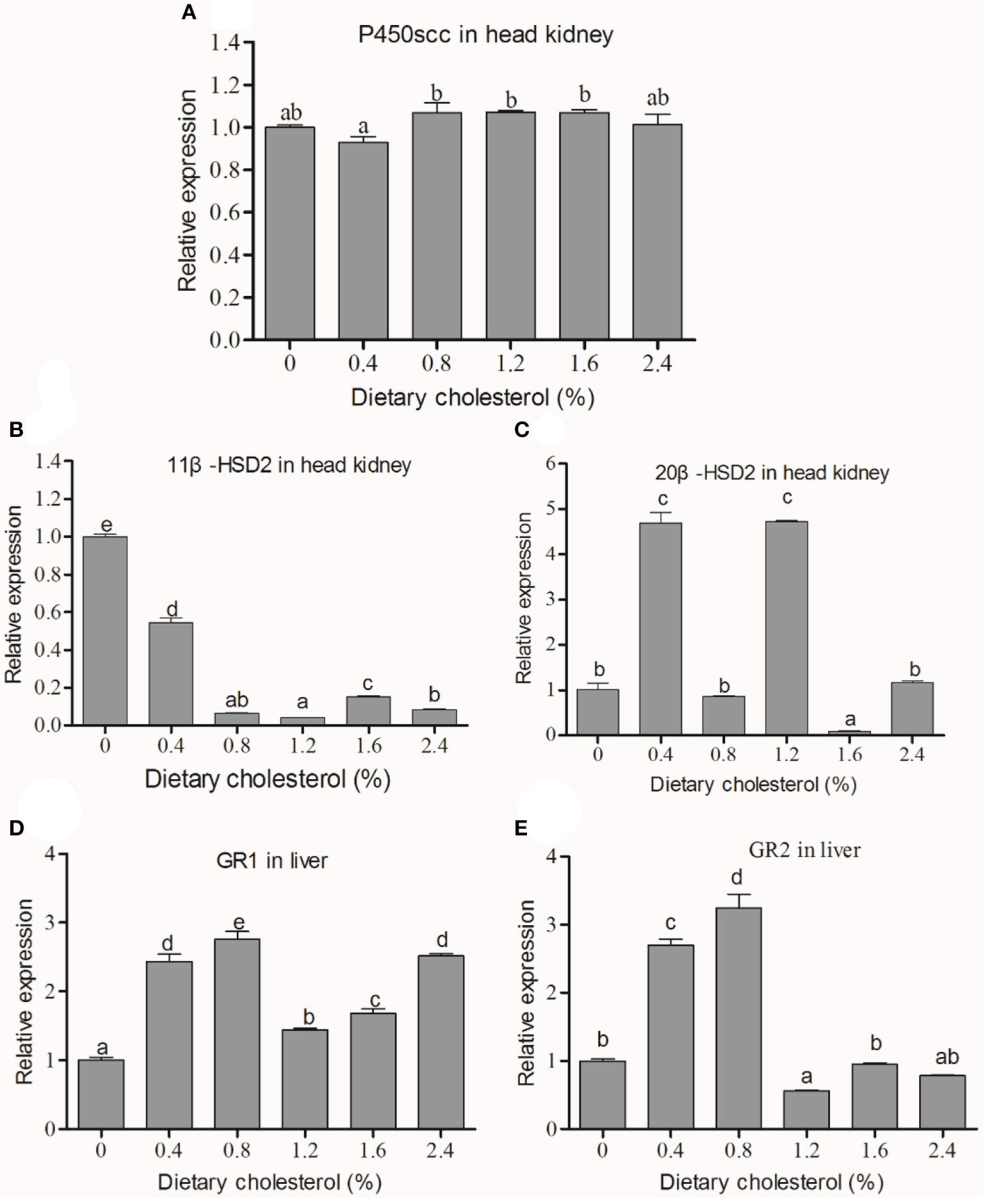
The mRNA expression of key genes in the HPI-axis. **(A)** The mRNA expression of P450scc in the head kidney; **(B)** the mRNA expression of 11β-HSD2 in the head kidney; **(C)** the mRNA expression of 20β-HSD2 in the head kidney; **(D)** the mRNA expression of GR1 in the liver; **(E)** the mRNA expression of GR2B in the liver. The abscissa indicates the diets, with the different cholesterol percentages labeled 0, 0.4, 0.8, 1.2, 1.6, and 2.4%. The ordinate represents the fold change in the relative gene expression of the three genes in different groups relative to the 0% cholesterol group. The different lowercase letters represent significant differences among groups (*P* < 0.05). Values (mean ± S.E.) of the mRNA expression levels are the mean of nine fish.

The P450scc mRNA level in the head kidney in the 0.4% group was significantly lower than those in the 0.8%, 1.2% and 1.6% groups (*P* < 0.05) (Figure 2A). However, no significant difference was found between the cholesterol-supplemented groups and the control (*P* > 0.05). The expression of 11β-HSD2 mRNA expressions in the head kidney decreased with increasing cholesterol (*P* < 0.05), and the control group had the highest expression of 11β-HSD2 (Figure 2B). The 20β-HSD2 mRNA expressions in the 0.4 and 1.2% groups were significantly higher than those in the other groups (Figure 2C). The GR1 mRNA expression in the liver initially increased with increasing dietary cholesterol, reaching the maximum value in the 0.8% cholesterol group, and then they decreased in the 1.2% cholesterol group before increasing again and reaching a second peak in the 2.4% cholesterol group (Figure 2D). The GR2B mRNA expression in the liver initially increased with the increase in cholesterol, reaching the highest value in the 0.8% cholesterol group, but it significantly dropped when the dietary cholesterol exceeded 0.8% (*P* < 0.05) (Figure 2E).

### The activity of Na^+^/K^+^-ATPase and the content of cortisol

**Figure 3:**
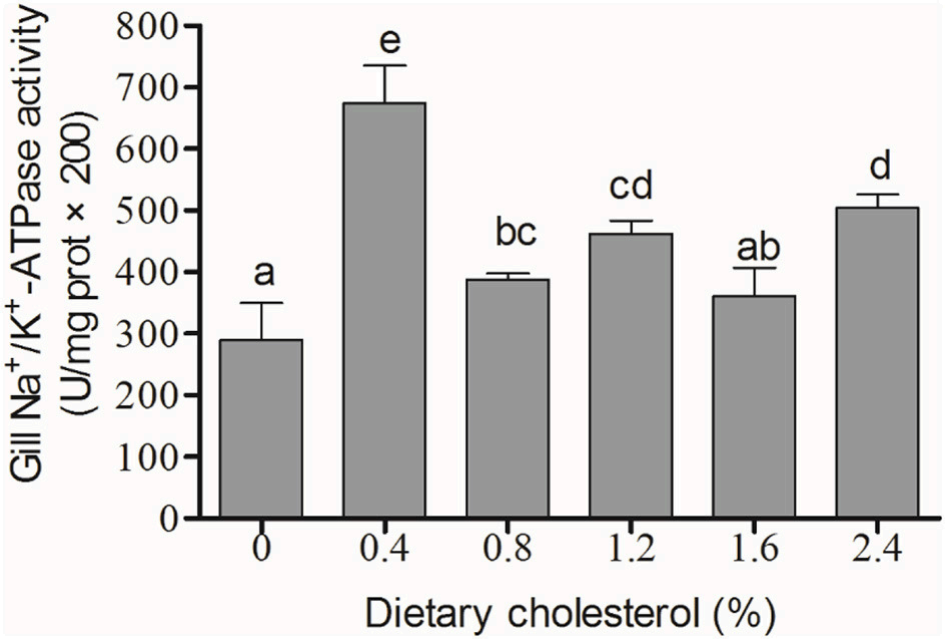
The activity of Na^+^/K^+^-ATPase in the gill. The abscissa indicates the diets, with the different cholesterol percentages labeled as 0, 0.4, 0.8, 1.2, 1.6, and 2.4%. The ordinate represents the activity of Na^+^/K^+^-ATPase, and the units are U/(mg protein). The different lowercase letters represent significant differences among groups (*P* < 0.05). Values (mean ± S.E.) of the Na^+^/K^+^-ATPase activity are the mean of nine fish.

**Figure 4:**
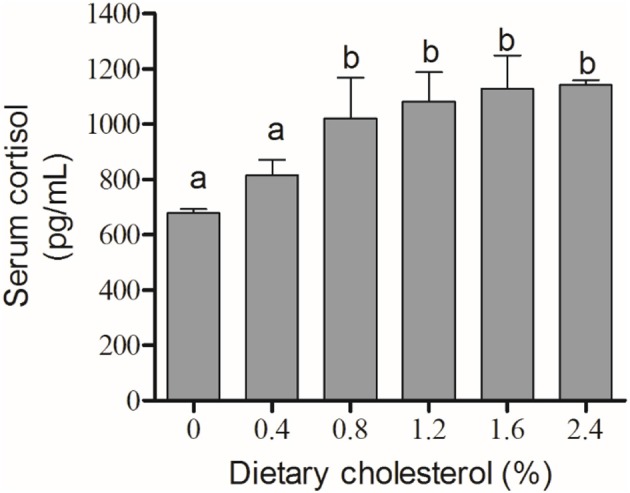
The content of cortisol in the serum. The abscissa indicates the diets, with the different cholesterol percentages labeled 0, 0.4, 0.8, 1.2, 1.6, and 2.4%. The ordinate represents the content of cortisol, and the units are pg/mL. The different lowercase letters represent significant differences among groups (*P* < 0.05). Values (mean ± S.E.) of the cortisol content in the serum are the mean of nine fish.

The activity of Na^+^/K^+^-ATPase in the gill filaments was significantly elevated by the increase in cholesterol in the 0.4, 0.8, 1.2, and 2.4% groups compared with the control group, and it achieved the highest level in the 4% group (*P* < 0.05) (Figure 3). The concentration of cortisol in the serum initially increased significantly with increasing levels of dietary cholesterol, but it leveled off when the cholesterol level exceeded 0.8% (*P* < 0.05) (Figure 4).

## Discussion

This is the first report on the effects of dietary cholesterol on the growth performance and osmoregulation of Nile tilapia in a brackish water environment. In this study, the weight gain of fish fed 0.4% cholesterol was higher than those fed other diets with cholesterol supplementation, but it did not differ from that of fish fed the control diet. These findings indicate that dietary cholesterol supplementation does not substantially improve the growth of Nile tilapia, and high cholesterol in their diet could lead to various negative effects. Similarly, other studies have also found that cholesterol supplementation to a diet containing high plant-derived protein does not significantly improve the survival of juvenile turbot *Scophthalmus maximus* L. (Yun et al., 2011), rainbow trout *O. mykiss* (Deng et al., 2013a, 2014) and orange-spotted grouper *Epinephelus coioides* (Zhang et al., 2016). Similarly, dietary cholesterol had no substantial beneficial effects on growth or body composition of juvenile hybrid striped bass (Sealey et al., 2001). The possible explanation for this is that bony fish have the capacity of *de novo* cholesterol synthesis from acetate (Bjerkeng et al., 1999; Deng et al., 2010). In mud crab *Scylla serrata*, megalopa showed highest survival fed diet with 0.8% cholesterol (Holme et al., 2006). Similar to our study, no benefits form cholesterol supplementation in excess of the dietary requirement were observed when compared to the basal diet under low salinity water (Roy et al., 2006).

Growth inhibition was found in fish fed > 0.8% cholesterol. This result may be related to the inhibition of *de novo* synthesis by converting cholesterol to bile acids through the up-regulation of cholesterol 7α-hydroxylase activity and the down-regulation of 3-hydroxy-3-methylglutaryl-CoA reductase activity in fish (Yun et al., 2011; Deng et al., 2013b). Meanwhile, negative effects on growth by excessive dietary cholesterol supplementation may be related to lipid metabolism disorder in the liver and serum of Nile tilapia. Dietary cholesterol directly affected the cholesterol contents in the serum and liver of Nile tilapia. The serum LDL-C content significantly increased with the increase in serum cholesterol in our study. LDL transfers cholesterol from the liver to the peripheral tissues (Deng et al., 2010). It is well known that serum components such as total cholesterol and LDL-C play an important role in health, immunity and antioxidant capabilities; therefore, the dynamic balance of these substances is very important to the health of organisms (Zhu et al., 2003; Hui et al., 2012). A significant increase in the contents of cholesterol and LDL-C in the serum may cause lipid metabolism disorder and induce hyperlipidemia (Ye et al., 2011; Zhai and Liu, 2013). Similarly, *Scylla serrate* showed a higher weight gain when fed 0.5–0.79% dietary cholesterol, and diets containing more than 1.12% cholesterol had an adverse effect on growth performance (Sheen, 2000). Therefore, excess dietary cholesterol supplements can increases in serum cholesterol and LDL-C contents, which may further affect the health of Nile tilapia cultivated in brackish water.

During the synthesis of cortisol from cholesterol, the HPI axis plays an important role in signal transduction and activation. The HPI axis in teleost fish is comparable to the mammalian stress axis (hypothalamus-pituitary-adrenal; HPA) as a result of convergent evolution (Bonga and Wendelaar, 1997; Mommsen et al., 1999), and it is also important in stress regulation and adaptation and acclimation for fish in a dynamic environment. As an effector of the HPI-axis, cortisol is the major stress hormone regulating a variety of metabolic processes and mediating the appropriate fight-or-flight response (Bonga and Wendelaar, 1997; Mommsen et al., 1999; McCormick et al., 2008). The significantly higher mRNA expression levels of P450scc in Nile tilapia fed 0.8, 1.2, and 1.6% cholesterol than those in Nile tilapia fed 0.4% cholesterol corresponds to the increase in serum cortisol in the Nile tilapia in our study. Meanwhile, the mRNA expression levels of 11β-HSD2 significantly decreased with the increase in the dietary cholesterol supplement. The 11β-HSD gene catalyzes the intracellular interconversion between hormonally active cortisol and inactive cortisone (Sandeep and Walker, 2001; Seckl and Walker, 2001; Ishii et al., 2007), and the 11β-HSD2 gene catalyzes the conversion of cortisol to cortisone (Tomlinson et al., 2004). The significant decrease in 11β-HSD2 mRNA expression in the serum and liver due to the increasing cholesterol levels is related to the negative feedback of cortisol production (Kusakabe et al., 2003; Terao et al., 2013). The 20β-HSD2 gene plays a role in further catabolic steps of cortisol inactivation, and it catalyzes the conversion of cortisone to 20β-hydroxycortisone (Tokarz et al., 2012). However, in the present study, the mRNA expression of the 20β-HSD2 gene in the head kidney showed fluctuating changes with the increase in dietary cholesterol. Thus, the different mRNA expression tendencies of two crucial genes involved in cortisol inactivation and excretion indicate a modest increase in cortisol in the serum of Nile tilapia fed different levels of cholesterol.

Chronic stress can increase the cortisol level after an exposure period of hours, days, or weeks (Mcewen, 2008). The stress-induced cortisol response mediated by the GR is a key signal orchestrating metabolic adjustments that are critical to cope with the enhanced energy demand for homeostasis due to stress (Sathiyaa and Vijayan, 2003). Teleost fish have two different GR paralogs, GR1 and GR2 (Prunet et al., 2006; Schaaf et al., 2008), which are involved in the negative feedback loop of circulating cortisol levels in response to elevated steroids (Bonga and Wendelaar, 1997; Mommsen et al., 1999). In Atlantic salmon, cortisol can regulate ion transporter mRNA expression levels in fresh water by signaling mediated via the GR (Kiilerich et al., 2007). The up-regulation of GR1 and GR2 mRNA in the 0.4 and 0.8% cholesterol groups compared with the control group reflects the positive regulation of cortisol contents in Nile tilapia, as reported in rainbow trout (*O. mykiss*) (Teles et al., 2013). High cholesterol in the serum of Nile tilapia fed a ≥ 1.2% cholesterol supplement can induce the expression of GR1 and GR2 mRNA in the liver due to the negative feedback regulation mechanism in teleost fish (Barton, 2002). Although GR2 is more sensitive to cortisol than GR1, it has a higher transactivation activity at a lower cortisol level than GR1 (Bury et al., 2003). Nevertheless, the functional difference between GR1 and GR2 is still poorly understood (Bury et al., 2003; Prunet et al., 2006; Li et al., 2012). More research should be done to explore the relationship between the GR and salinity stress.

As an effector of the HPI axis, cortisol levels in the serum are typically measured to determine the stress condition of individuals (Ramsay et al., 2006). Plasma cortisol and osmolality in tilapia changed rapidly in response to salinity stress (Kammerer et al., 2010). Tilapia injected with cortisol can increase the number of ion-transporting chloride cells (Foskett et al., 1981) and the Na^+^/K^+^-ATPase activity in gills to cope with osmotic stress (Dang et al., 2000; McCormick et al., 2008). However, long-term exposure to cortisol leads to a series of negative effects on fish (Gilmour et al., 2005), including the reduction of cortisol sensitivity to other acute stresses (Overli et al., 1999; Sloman et al., 2002; Jeffrey et al., 2014). In our study, a slight increase in cortisol induced by feeding fish with a 0.4% cholesterol supplement significantly increased the activity of Na^+^/K^+^-ATPase in the gills of Nile tilapia. The gill activities of Na^+^/K^+^ ATPase in fish fed the 0.8, 1.2, and 2.4% cholesterol supplement diets were significantly higher than that of the control fish, although they were still lower than the activity in the gills of fish fed the 0.4% cholesterol supplement diet. Previous studies have shown that the chronic exposure of fish to high cortisol could change energy metabolism (Gilmour et al., 2012), down-regulate energy storage (Barton et al., 1987; Gilmour et al., 2012) and slow growth rates (Dill, 1989; DiBattista et al., 2006). In Atlantic salmon, a cortisol treatment resulted in increased physiological levels of cortisol, increased gill Na^+^/K^+^ ATPase activity and improved salinity tolerance (McCormick, 1996; McCormick et al., 2008). Therefore, the increase in cortisol induced by feeding fish with a 0.4% dietary cholesterol supplement may be an appropriate amount for the osmoregulation of Nile tilapia under hyperosmotic stress.

## Conclusions

Under chronic hyperosmotic stress, cholesterol is not an essential requirement for Nile tilapia, but it can be used as a feed nutrient additive. The HPI-axis and related genes, such as 11β-HSD2, 20β-HSD2, GR1 and GR2, play an important role in the balance of serum cortisol, although excessive cholesterol was supplemented in the diet of Nile tilapia under salinity stress. The proper concentration of dietary cholesterol (~0.4%) can contribute to the osmoregulation of Nile tilapia under salinity stress.

## Author contributions

CX, EL, ZX, LC, and XW designed the research. CX, ZX, and YS conducted the research and contributed to the data acquisition and analysis. CX, ZX, and EL contributed to the draft and the final writing of the paper. ML, JQ, LC, and XW revised the paper critically. All authors agree to be accountable for all aspects of the work and have approved the final manuscript.

### Conflict of interest statement

ML was employed by company Hainan Dingda Aquaculture Co., Ltd. The other authors declare that the research was conducted in the absence of any commercial or financial relationships that could be construed as a potential conflict of interest.

## References

[B1] Alsop D. Vijayan M. (2009). The zebrafish stress axis: molecular fallout from the teleost-specific genome duplication event. Gen. Comp. Endocrinol. 161, 62–66. 10.1016/j.ygcen.2008.09.011 18930731

[B2] Aluru N. Vijayan M. M. (2006). Aryl hydrocarbon receptor activation impairs cortisol response to stress in rainbow trout by disrupting the rate-limiting steps in steroidogenesis. Endocrinology 147, 1895–1903. 10.1210/en.2005-1143 16410306

[B3] Aluru N. Vijayan M. M. (2008). Molecular characterization, tissue-specific expression, and regulation of melanocortin 2 receptor in rainbow trout. Endocrinology 149, 4577–4588. 10.1210/en.2008-0435 18535097 PMC2553378

[B4] Barton B. A. (2002). Stress in fishes: a diversity of responses with particular reference to changes in circulating corticosteroids. Integr. Comp. Biol. 42, 517–525. 10.1093/icb/42.3.517 21708747

[B5] Barton B. A. Schreck C. B. Barton L. D. (1987). Effects of chronic cortisol administration and daily acute stress on growth, physiological conditions, and stress responses in juvenile rainbow trout. Dis. Aquat. Org. 2, 173–185. 10.3354/dao002173

[B6] Bełtowski J. Wójcicka G. (2002). Spectrophotometric method for the determination of renal ouabain-sensitive H^+^, K^+^-ATPase activity. Acta Biochim. Pol. 49, 515–527. 12362994

[B7] Bjerkeng B. Storebakken T. Wathne E. (1999). Cholesterol and short-chain fatty acids in diets for Atlantic salmon *Salmo salar* (L.): effects on growth, organ indices, macronutrient digestibility, and fatty acid composition. Aquacul Nutr. 5, 181–191. 10.1046/j.1365-2095.1999.00103.x

[B8] Bonga S. Wendelaar E. (1997). The stress response in fish. Physiol. Rev. 77, 591–625. 10.1152/physrev.1997.77.3.591 9234959

[B9] Bury N. R. Sturm A. Le Rouzic P. Lethimonier C. Ducouret B. Guiguen Y.. (2003). Evidence for two distinct functional glucocorticoid receptors in teleost fish. J. Mol. Endocrinol. 31, 141–156. 10.1677/jme.0.0310141 12914532

[B10] Chen J. (2006). Effects of Fermentation, Exogenous Enzyme and Feeding Stimulants on Utilization of Soybean Meal Protein by Japanese Flounder (Paralichthys olivaceus). Ph D thesis, Ocean University.

[B11] Dang Z. Balm P. H. Flik G. Wendelaar Bonga S. E. Lock R. A. (2000). Cortisol increases Na^+^/K^+^-ATPase density in plasma membranes of gill chloride cells in the freshwater tilapia *Oreochromis mossambicus*. J. Exp. Biol. 203, 2349–2355. 10887073 10.1242/jeb.203.15.2349

[B12] Deng J. Bi B. Kang B. Kong L. Wang Q. Zhang X. (2013b). Improving the growth performance and cholesterol metabolism of rainbow trout (*Oncorhynchus mykiss*) fed soyabean meal-based diets using dietary cholesterol supplementation. Br. J. Nutr. 110, 29–39. 10.1017/S0007114512004680 23182370

[B13] Deng J. Mai K. Ai Q. Zhang W. Wang X. Tan B. (2010). Interactive effects of dietary cholesterol and protein sources on growth performance and cholesterol metabolism of Japanese flounder (*Paralichthys olivaceus*). Aquacult Nutr. 16, 419–429. 10.1111/j.1365-2095.2009.00681.x

[B14] Deng J. Kang B. Tao L. Rong H. Zhang X. (2013a). Effects of dietary cholesterol on antioxidant capacity, non-specific immune response, and resistance to *Aeromonas hydrophila* in rainbow trout (*Oncorhynchus mykiss*) fed soybean meal-based diets. Fish Shellfish Immun. 34, 324–331. 10.1016/j.fsi.2012.11.008 23207478

[B15] Deng J. Zhang X. Long X. Tao L. Wang Z. Niu G. (2014). Effects of dietary cholesterol supplementation on growth and cholesterol metabolism of rainbow trout (*Oncorhynchus mykiss*) fed diets with cottonseed meal or rapeseed meal. Fish Physiol. Biochem. 40, 1827–1838. 10.1007/s10695-014-9971-2 25119853

[B16] DiBattista J. D. Levesque H. M. Moon T. W. Gilmour K. M. (2006). Growth depression in socially subordinate rainbow trout *Oncorhynchus mykiss*: more than a fasting effect. Physiol. Biochem. Zool. 179, 675–687. 10.1086/504612 16826494

[B17] Dill L. M. (1989). The Relative growth of dominant and subordinate juvenile steelhead trout (*Salmo gairdneri*) fed equal rations. Behaviour 108, 104–113. 10.1163/156853989X00079

[B18] El-Zaeem S. Y. Ahmed M. M. M. Salama M. E. S. El-Maremie H. A. R. (2011). Production of salinity tolerant Nile tilapia, *Oreochromis niloticus* through traditional and modern breeding methods: II. Application of genetically modified breeding by introducing foreign DNA into fish gonads. Afr. J. Biotechnol. 10, 684–695. 10.13140/2.1.4462.4007

[B19] Engelhardt D. Dörr G. Jaspers C. Knorr D. (1985). Ketoconazole blocks cortisol secretion in man by inhibition of adrenal 11β-hydroxylase. Klin. Wochenschr. 63, 607–612. 10.1007/BF01733014 2993735

[B20] Fang L. S. Shufen C. (1989). Effect of salinity on the activities of digestive proteases from the tilapia fish, *Oreochromis niloticus* in different culture environments. Comp. Biochem. Phys. A 93, 439–443. 10.1016/0300-9629(89)90063-7

[B21] Fast A. W. Boyd C. E. (1992). Water circulation, aeration and other management practices, in Marine Shrimp Culture: Principles and Practices, eds Fast A. W. Lester L. J. (The Hague: Elsevier Science Publishers), 457–495.

[B22] Flik G. Klaren P. H. Van den Burg E. H. Metz J. R. Huising M. O. (2006). CRF and stress in fish. Gen. Comp. Endocr. 146, 36–44. 10.1016/j.ygcen.2005.11.005 16403502

[B23] Foskett J. K. Logsdon C. D. Turner T. Machen T. E. Bern H. A. (1981). Differentiation of the chloride extrusion mechanism during seawater adaption of a teleost fish, the cichlid *Sarotherodon mossambicus*. J. Exp. Biol. 93, 209–224.

[B24] Gilmour K. M. Dibattista J. D. Thomas J. B. (2005). Physiological causes and consequences of social status in salmonid fish. Integr. Comp. Biol. 45, 263–273. 10.1093/icb/45.2.263 21676770

[B25] Gilmour K. M. Kirkpatrick S. Massarsky A. Pearce B. Saliba S. Stephany C. É. (2012). The influence of social status on hepatic glucose metabolism in rainbow trout *Oncorhynchus mykiss*. Physiol. Biochem. Zool. 85:309 10.1086/666497 22705482

[B26] Gold P. W. Drevets W. C. Charney D. S. (2002). New insights into the role of cortisol and the glucocorticoid receptor in severe depression. Biol. Psychiat. 52, 381–385. 10.1016/S0006-3223(02)01480-4 12242053

[B27] Han B. (2013). Effects of Dietary Phospholipid, Cholesterol and Their Interactions on Growth Performance, Digestive Enzymes and Expression of Related Genes of Tongue Sole (Cynoglossus Semilaevis Günther) Larvae. Master thesis. Ocean University.

[B28] Holme M. H. Zeng C. Southgate P. C. (2006). The effects of supplemental dietary cholesterol on growth, development and survival of mud crab, *Scylla serrata*, megalopa fed semi-purified diets. Aquaculture 261, 1328–1334. 10.1016/j.aquaculture.2006.08.032

[B29] Hui F. U. Wang Q. K. Yun-Hai H. E. Ren D. D. (2012). Functional effect of dietary fiber from seaweed *Costaria costata* residues on reduce in serum lipids in mice. J. Dalian Ocean University 27, 200–204. 10.3969/j.issn.1000-9957.2012.03.002

[B30] Huising M. O. van Schooten C. Taverne-Thiele A. Hermsen T. Verburg-van Kemenade B. (2004). Structural characterisation of a cyprinid (*Cyprinus carpio* L.) CRH, CRH-BP and CRH-R1, and the role of these proteins in the acute stress response. J. Mol. Endocrinol. 32, 627–648. 10.1677/jme.0.0320627 15171705

[B31] Ishii T. Masuzaki H. Tanaka T. Arai N. Yasue S. Kobayashi N.. (2007). Augmentation of 11beta-hydroxysteroid dehydrogenase type 1 in LPS-activated J774.1 macrophages–role of 11beta-HSD1 in pro-inflammatory properties in macrophages. FEBS Lett. 581, 349–354. 10.1016/j.febslet.2006.11.032 17239856

[B32] Ismaili N. Garabedian M. J. (2004). Modulation of glucocorticoid receptor function via phosphorylation. Ann. N. Y Acad. Sci. 1024, 86–101. 10.1196/annals.1321.007 15265775

[B33] Jeffrey J. D. Gollock M. J. Gilmour K. M. (2014). Social stress modulates the cortisol response to an acute stressor in rainbow trout (*Oncorhynchus mykiss*). Gen Comp Endocr. 196, 8–16. 10.1016/j.ygcen.2013.11.010 24269985

[B34] Jobling M. (2011). National research council; nutrient requirements of fish. Aquacul Int. 20, 601–602. 10.1007/s10499-011-9480-6

[B35] Kammerer B. D. Cech J. J. Jr. Kültz D. (2010). Rapid changes in plasma cortisol, osmolality, and respiration in response to salinity stress in tilapia *(Oreochromis mossambicus)*. Comp. Biochem. Phys. A 157, 260–265. 10.1016/j.cbpa.2010.07.009 20647048

[B36] Kiilerich P. Kristiansen K. Madsen S. S. (2007). Cortisol regulation of ion transporter mRNA in *Atlantic salmon* gill and the effect of salinity on the signaling pathway. J. Endocrinol. 194, 417–427. 10.1677/JOE-07-0185 17641289

[B37] Kusakabe M. Nakamura I. Young G. (2003). Enzymatic activity of 11β-hydroxysteroid dehydrogenase in rainbow trout (*Oncorhynchus mykiss*). Fish Physiol. Biochem. 28, 197–198. 10.1023/B:FISH.0000030526.42177.8b

[B38] Lemarié G. Baroiller J. F. Clota F. Lazard J. Dosdat A. (2004). A simple test to estimate the salinity resistance of fish with specific application to *O. niloticus* and *S. melanotheron*. Aquaculture 240, 575–587. 10.1016/j.aquaculture.2004.07.014

[B39] Li X. Li X. Leng X. Liu X. M. Wang X. C. Li J. L. (2007). Effect of different salinities on growth and flesh quality of *Ctenopharyngodon idellus*. J Fish. China 3:010 10.3321/j.issn:1000-0615.2007.03.011

[B40] Li Y. Sturm A. Cunningham P. Bury N. R. (2012). Evidence for a divergence in function between two glucocorticoid receptors from a basal teleost. BMC Evol. Biol. 12:137 10.1186/1471-2148-12-137 22862956 PMC3457903

[B41] Long X. Wang Q. Han X. Zhang X. Deng J. (2013). Research advances on the mechanism of growth-promoting effects of dietary cholesterol in soybean meal-based diets of fish. J. Anhui Agr. Sci. 2954–2955. 10.3969/j.issn.0517-6611.2013.07.054

[B42] McCormick S. D. (1996). Effects of growth hormone and insulin-like growth factor I on salinity tolerance and gill Na^+^, K^+^-ATPase in Atlantic Salmon *(Salmo salar)*: interaction with cortisol. Gen. Comp. Endocr. 101, 3–11. 10.1006/gcen.1996.0002 8713639

[B43] McCormick S. D. Regish A. O'Dea M. F. Shrimpton J. M. (2008). Are we missing a mineralocorticoid in teleost fish? Effects of cortisol, deoxycorticosterone and aldosterone on osmoregulation, gill Na^+^, K^+^-ATPase activity and isoform mRNA levels in *Atlantic salmon*. Gen. Comp. Endocr. 157, 35–40. 10.1016/j.ygcen.2008.03.024 18462736

[B44] Mcewen B. S. (2008). Central effects of stress hormones in health and disease: understanding the protective and damaging effects of stress and stress mediators. Eur. J. Pharmacol. 583, 174–185. 10.1016/j.ejphar.2007.11.071 18282566 PMC2474765

[B45] Metz J. R. Huising M. O. Meek J. Taverne-Thiele A. J. Wendelaar Bonga S. E. Flik G. (2004). Localization, expression and control of adrenocorticotropic hormone in the nucleus preopticus and pituitary gland of common carp (*Cyprinus carpio* L.). J. Endocrinol. 182, 23–31. 10.1677/joe.0.1820023 15225128

[B46] Mommsen T. P. Vijayan M. M. Moon T. W. (1999). Cortisol in teleosts: dynamics, mechanisms of action, and metabolic regulation. Rev. Fish. Biol. Fisher. 9, 211–268. 10.1023/A:1008924418720

[B47] Mormède P. Andanson S. Aupérin B. Beerda B. Guémené D. Malnikvist J.. (2007). Exploration of the hypothalamic-pituitary-adrenal function as a tool to evaluate animal welfare. Physiol. Behav. 92, 317–339. 10.1016/j.physbeh.2006.12.003 17234221

[B48] Nauck M. Warnick G. R. Rifai N. (2002). Methods for measurement of LDL-cholesterol: a critical assessment of direct measurement by homogeneous assays versus calculation. Clin. Chem. 48, 236–254. 11805004

[B49] Ntabo J. K. (2012). Tolerance, Survival Rate and Growth of Tilapia (Oreochromis niloticus) Fingerlings at Different Salinity Levels. Rep Ocean Public.

[B50] Overli O. Olsen R. E. Lovik F. Ringo E. (1999). Dominance hierarchies in Arctic charr, *Salvelinus alpinus* L.: differential cortisol profiles of dominant and subordinate individuals after handling stress. Aquac Res. 30, 259–264. 10.1046/j.1365-2109.1999.00322.x

[B51] Peckett A. J. Wright D. C. Riddell M. C. (2011). The effects of glucocorticoids on adipose tissue lipid metabolism. Metab. Clin. Exp. 60, 1500–1510. 10.1016/j.metabol.2011.06.012 21864867

[B52] Peres H. Lim C. Klesius P. H. (2003). Nutritional value of heat-treated soybean meal for channel catfish (*Ictalurus punctatus*). Aquaculture 225, 67–82. 10.1016/S0044-8486(03)00289-8

[B53] Prunet P. Sturm A. Milla S. (2006). Multiple corticosteroid receptors in fish: from old ideas to new concepts. Gen. Comp. Endocr. 147, 17–23. 10.1016/j.ygcen.2006.01.015 16545810

[B54] Ramsay J. M. Feist G. W. Varga Z. M. Westerfield M. Kent M. L. Schreck C. B. (2006). Whole-body cortisol is an indicator of crowding stress in adult zebrafish, *Danio rerio*. Aquaculture 258, 565–574. 10.1016/j.aquaculture.2006.04.020

[B55] Roy L. A. Davis D. A. Saoud I. P. (2006). Effects of lecithin and cholesterol supplementation to practical diets for *Litopenaeus vannamei* reared in low salinity waters. Aquaculture 257, 446–452. 10.1016/j.aquaculture.2006.02.059

[B56] Sandeep T. C. Walker B. R. (2001). Pathophysiology of modulation of local glucocorticoid levels by 11β-hydroxysteroid dehydrogenases. Trends Endocrin. Met. Tem. 12, 446–453. 10.1016/S1043-2760(01)00499-4 11701343

[B57] Sathiyaa R. Vijayan M. M. (2003). Autoregulation of glucocorticoid receptor by cortisol in rainbow trout hepatocytes. Am. J. Physiol. Cell. 284:C1508. 10.1152/ajpcell.00448.2002 12584114

[B58] Schaaf M. J. Champagne D. van Laanen I. H. van Wijk D. C. Meijer A. H. Meijer O. C.. (2008). Discovery of a functional glucocorticoid receptor beta-isoform in zebrafish. Endocrinology 149, 1591–1599. 10.1210/en.2007-1364 18096659

[B59] Schmittgen T. D. Livak K. J. (2008). Analyzing real-time PCR data by the comparative CT method. Nat. Protoc. 3, 1101–1108. 10.1038/nprot.2008.73 18546601

[B60] Sealey W. M. Craig S. R. Dmiii G. (2001). Dietary cholesterol and lecithin have limited effects on growth and body composition of hybrid striped bass (*Morone chrysops x M. saxatilis)*. Aquacult. Nutr. 7, 25–31. 10.1046/j.1365-2095.2001.00159.x

[B61] Seckl J. R. Walker B. R. (2001). Minireview: 11beta-hydroxysteroid dehydrogenase type 1- a tissue-specific amplifier of glucocorticoid action. Endocrinology 142, 1371–1376. 10.1210/endo.142.4.8114 11250914

[B62] Seidelin M. Madsen S. S. (1999). Endocrine control of Na^+^, K^+^-ATPase and chloride cell development in brown trout (*Salmo trutta*). J. Endocrinol. 162, 127–135. 10.1677/joe.0.1620127 10396029

[B63] Sheen S. S. (2000). Dietary cholesterol requirement of juvenile mud crab Scylla serrate. Aquaculture 189, 277–285. 10.1016/S0044-8486(00)00379-3

[B64] Sheen S. S. Liu P. C. Chen S. N. Chen J. C. (1994). Cholesterol requirement of juvenile tiger shrimp (*Penaeus monodon*). Aquaculture 125, 131–137. 10.1016/0044-8486(94)90289-5

[B65] Sloman K. A. Montpetit C. J. Gilmour K. M. (2002). Modulation of catecholamine release and cortisol secretion by social interactions in the rainbow trout, *Oncorhynchus mykiss*. Gen. Comp. Endocr. 127:136 10.1016/S0016-6480(02)00033-3 12383441

[B66] Steffens W. (1989). Principles of Fish Nutrition. New York, NY; Chichester; Brisbane, QLD; Toronto, ON: Ellis Horwood; Halsted Press; John Wiley.

[B67] Teles M. Tridico R. Callol A. Fierro-Castro C. Tort L. (2013). Differential expression of the corticosteroid receptors GR1, GR2 and MR in rainbow trout organs with slow release cortisol implants. Comp. Biochem. Physiol. Part A Mol. Integr. Physiol. 164, 506–511. 10.1016/j.cbpa.2012.12.018 23277222

[B68] Terao M. Itoi S. Murota H. Katayama I. (2013). Expression profiles of cortisol-inactivating enzyme, 11β-hydroxysteroid dehydrogenase-2, in human epidermal tumors and its role in keratinocyte proliferation. Exp. Dermatol. 22, 98–101. 10.1111/exd.12075 23362866

[B69] Tokarz J. Mindnich R. Norton W. Möller G. Hrabé de Angelis M. Adamski J. (2012). Discovery of a novel enzyme mediating glucocorticoid catabolism in fish: 20beta-hydroxysteroid dehydrogenase type 2. Mol. Cell. Endocrinol. 349, 202–213. 10.1016/j.mce.2011.10.022 22061621

[B70] Tomlinson J. W. Walker E. A. Bujalska I. J. Draper N. Lavery G. G. Cooper M. S.. (2004). 11beta-hydroxysteroid dehydrogenase type 1: a tissue-specific regulator of glucocorticoid response. Endocr. Rev. 25, 831–866. 10.1210/er.2003-0031 15466942

[B71] Twibell R. G. Wilson R. P. (2004). Preliminary evidence that cholesterol improves growth and feed intake of soybean meal-based diets in aquaria studies with juvenile channel catfish, *Ictalurus punctatus*. Aquaculture 236, 539–546. 10.1016/j.aquaculture.2003.10.028

[B72] Xu Z. Gan L. Li T. Xu C. Chen K. Wang X.. (2015). Transcriptome profiling and molecular pathway analysis of genes in association with salinity adaptation in Nile tilapia *Oreochromis niloticus*. PLoS ONE 10:e0136506. 10.1371/journal.pone.0136506 26305564 PMC4548949

[B73] Yang C. G. Wang X. L. Tian J. Liu W. Wu F. Jiang M.. (2013). Evaluation of reference genes for quantitative real-time RT-PCR analysis of gene expression in Nile tilapia (*Oreochromis niloticus*). Gene 527, 183–192. 10.1016/j.gene.2013.06.013 23792389

[B74] Ye J. Liu X. Wang Z. Wang K. (2011). Effect of partial fish meal replacement by soybean meal on the growth performance and biochemical indices of juvenile Japanese flounder *Paralichthys olivaceus*. Aquacul Int. 19, 143–153. 10.1007/s10499-010-9348-1

[B75] Yun B. Mai K. Zhang W. Xu W. (2011). Effects of dietary cholesterol on growth performance, feed intake and cholesterol metabolism in juvenile turbot (*Scophthalmus maximus* L.) fed high plant protein diets. Aquaculture 319, 105–110. 10.1016/j.aquaculture.2011.06.028

[B76] Zhai S. W. Liu S. L. (2013). Effects of dietary quercetin on growth performance, serum lipids level and body composition of tilapia (*Oreochromis niloticus*). Ital J. Anim. Sci. 12:e85. 10.4081/ijas.2013.e85

[B77] Zhang W. C. Dong X. H. Tan B. P. Zhang S. Chi S. Y. Yang Q. H. (2016). Effects of dietary cholesterol content on growth performance, tissue biochemical indices and liver lipid metabolism related enzyme activities of orange-spotted grouper (*Epinephelus coioides*). Chin. J. Anim. Nutr. 28, 1945–1955. 10.3969/j.issn.1006-267x.2016.06.038

[B78] Zhu Z. Y. Gao C. Y. Huang K. J. Zhang J. Y. Chen Y. Niu Z. M. (2003). The relationship of low density lipoprotein-cholesterol/high density lipoprotein-cholesterol ratio, triglyceride/high density lipoprotein-cholesterol ratio to coronary heart disease. Chin Circ. J. 18, 273–275.

